# Complex Extraction of Metals in an Aqueous Two-Phase System Based on Poly(Ethylene Oxide) 1500 and Sodium Nitrate

**DOI:** 10.3390/molecules24224078

**Published:** 2019-11-11

**Authors:** Yulia A. Zakhodyaeva, Inna V. Zinov’eva, Elena S. Tokar, Andrey A. Voshkin

**Affiliations:** Kurnakov Institute of General and Inorganic Chemistry of the Russian Academy of Sciences, 31 Leninsky Prospect, Moscow 119991, Russia; yz@igic.ras.ru (Y.A.Z.); iz@igic.ras.ru (I.V.Z.); koltsova.e@inbox.ru (E.S.T.)

**Keywords:** aqueous two-phase systems, metal extraction, liquid-liquid equilibria, spent Ni-MH battery, e-waste, poly(ethylene oxide), sodium nitrate

## Abstract

This article presents an ecologically safe aqueous two-phase system based on poly(ethylene oxide) with a molecular weight of 1500, designed for complex extraction of Ni(II), Co(II), Fe(III), Mn(II), Zn(II), Cu(II), and Al(III) from nitrate solutions. A kinetic dependence has been investigated for a distribution ratio for the metals examined. The influence of pH-values, temperature, initial metal concentration, and nitric acid content have on the extraction of a wide range of metals in the heterogeneous poly(ethylene oxide) 1500-NaNO_3_-H_2_O system has been discovered. As a result, the complex extraction of metals (E_Me_ > 60%) was achieved in one step of extraction without introducing additional chemicals into the system.

## 1. Introduction

One of the most important issues in the world today is the growth of e-waste volumes [1,2], the growing quantity of which, first and foremost, is a result of constantly advancing technologies and the ever greater human desire to possess new, more modern technical means. Power supply elements (batteries, accumulator cells) are one of the types of e-wastes which often enter the environment. Among the most popular chemical sources of electrical energy nickel metal-hydrate (Ni-MH) batteries are worth special consideration [3]. The cylindrical Ni-MH batteries contain a large quantity of important metals (19.9% Ni, 15.4% Fe, 12.1% La, 5.16% Ce, 4.41% Co, 0.79% Zn, 0.73% Mn, 0.25% Al, 0.01% Cu, among others) [4]. Due to the economic value of metals contained in electronic equipment, developing an effective process for recycling waste is a priority objective in achieving “a minimization of waste” and “the stable recovery of resources” in accordance with the principles of “green chemistry”.

Over a number of years, different methods have been used for metal ion extraction. These methods have been ion exchange [5], adsorption [6], chemical precipitation [7], membrane separation [8], and solvent extraction [9,10,11]. However, most of the above methods come with certain disadvantages, such as high consumption of organic solvents, incomplete extraction of metal ions, and the high cost of the chemicals. Today, aqueous two-phase systems (ATPS) based on water-soluble polymers [12] and ionic liquids [13], which are actively studied for the elaboration of the extraction and purification processes of both organic [14] and inorganic substances [15], have a major chance of being put into a practical use. The most widely used systems are those formed by both a polymer and salt or by two polymers (usually by polyethylene glycol and dextran). Meanwhile, carbonates, phosphates, sulfates, fluorides, thiocyanates, iodides, perchlorates, etc., are used as phase-forming salts [16]. Under certain conditions, the polymer and inorganic salt can form a heterogeneous system with a high water content (over 80%). While the top phase is enriched with a polymer, the bottom phase is enriched with a phase-forming salt.

By now, a sufficient number of papers have been published on the extraction of non-ferrous, rare-earth, and precious metals in heterogeneous systems without organic solvents based on polymers, surface active agents, and ionic liquids [17,18,19,20]. The results of studies have been summarized in reviews [12,21]. In most cases, the metal is extracted from sulfate media using polymers, such as polyethylene glycol with molecular weights of 1500–6000; however, the extraction efficiency of some metals in such systems never amounts to 30% due to the formation of strong high-charge acid complexes [22]. To boost the extraction efficiency, additional inorganic (thiocyanates and iodides of alkali metals) [20,23,24] and organic complex forms (dithizone, PAN, Arsenazo III, etc.) [25,26] are introduced into such systems, which allow for the quantitative extraction of metal ions into a polymer phase). However, the introduction of most of these chemicals does not correspond to one of the principles of green chemistry, i.e., a reduction in the toxic and harmful substances used.

Thus, the task of searching for ecologically safe, heterogeneous systems with a high extraction capacity is quite an important issue.

Traditionally, sulfuric, hydrochloric, and nitric acid solutions have been used in metal ion leaching from power source elements [27]. For the first time, this article studied the extraction of Ni(II), Co(II), Fe(III), Mn(II), Zn(II), Cu(II), and Al(III) from nitrate solutions in the aqueous two-phase systems based on poly(ethylene oxide) (PEO) 1500 which we proposed earlier [28]. The purpose of this work is to establish the interphase distribution regularities for a number of metals (Ni, Co, Fe, Mn, Cu, Zn, Al) in the PEO 1500-NaNO_3_-H_2_O extraction system. The effect of various ATPS parameters (temperature, pH-value, initial metal concentration, etc.) on the efficiency of metal extraction was studied. Application of the proposed ATPS for the extraction of metals does not imply the use of organic solvents and allows them to be reused, which is compatible with the principles of green chemistry.

## 2. Results and Discussion

### 2.1. Determination of the Equilibrium Time in ATPS

**Figure 1:**
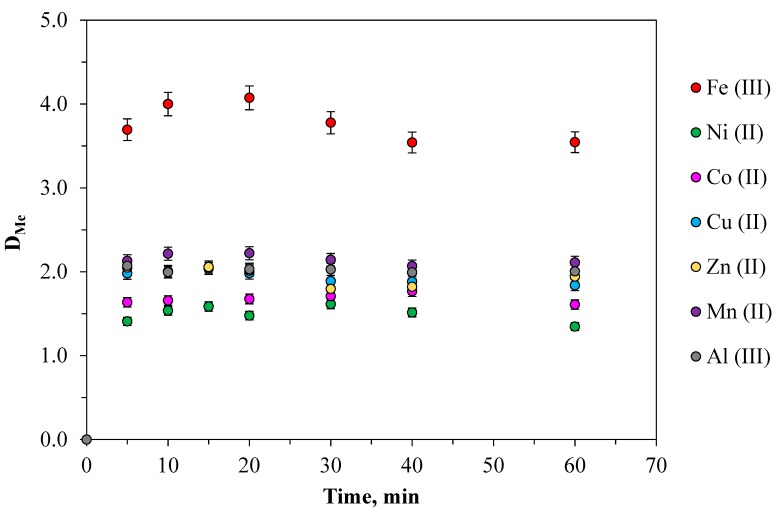
Effect of the phase contact time on the metal extraction in PEO 1500 (16.3 wt%)-NaNO_3_ (36.0 wt%)-H_2_O system: [Me]_in_ = 0.01 mol·L^−1^; the temperature was 298.15 K, the phase ratio: 1:1.

To obtain equilibrium data during extraction, kinetic relationships were established for all the metals studied in the PEO 1500 (16.3 wt%)-NaNO_3_ (36 wt%)-H_2_O system as shown in Figure 1. The experiment lasted for 5–60 min at a temperature of 298.15 K, [Me]_in_ = 0.01 mol·L^−1^. All the metals were extracted into the PEO-phase rather quickly. Equilibrium was reached in the nitrate systems in 5 min, which had to do with the low surface tension of ATPS and, as a result, via a quick mass transfer. Thus, the most favorable mixing time was over 5 min. However, with prolonged contact time, a slight decline could be seen for the distribution ratio, which might be caused by the effect of emulsification in long-time agitation. Increasing the time up to 60 min did not have a substantial impact on extraction recovery.

Preliminary studies showed that metals in the sulfate systems (e.g., PEO 1500-Na_2_SO_4_-H_2_O) could not be extracted due to the formation of strong high-charge [Me(SO_4_)_x_]^y−^ type acid complexes which are not extracted by water-soluble polymers [17,22,29]. The anionic sulphates [Me(SO_4_)_x_]^y−^ formed from the metal ion with a high negative hydration Gibbs energy (Δ*G*_hyd_) and the anion with a strong salting-out effect (${\Delta G}_{\mathrm{hyd}}{(\mathrm{SO}}_{4}^{2-})=-1145\;\mathrm{kJ}/\mathrm{mol}$) [30] have a lower affinity for the PEO-rich phase of the extraction system. Under these conditions the extraction efficiency can be changed by modifying the nature of the metals species by using an anion with lower hydration (for example Cl^−^), which will improve the selectivity of the extraction process [29].

In this paper, we achieved high metal extraction recovery values in the system based on sodium nitrate without introducing additional extraction agents.

Owing to strong hydration, metal ions in nitrate media are predominantly in cationic aquacomplex forms [Me(H_2_O)_x_]^n+^; their composition and content were determined by the pH of the solution, the concentration of nitrate ions, and the stability constant of the complex. ${\mathrm{NO}}_{3}^{-}$ -ions practically do not affect the hydrolysis of cations [31].

In the studied extraction system with PEO 1500 and NaNO_3_ compared to sulfate systems where the phase ratio is 1:1 the polymer phase contains a large amount of sodium nitrate with a low value of $\Delta G_{\mathrm{hyd}}{(\mathrm{NO}}_{3}^{-})=-275\;\mathrm{kJ}/\mathrm{mol}$ [30], commensurate with the amount of polymer (Table 1). In this case, hydrophilic cationic aquacomplexes formed by metals with high negative hydration Gibbs energy are predominantly distributed in the polymer phase, forming hydrogen bonds with polyethylene oxide molecules. The metal cation is in the same form in the initial solution and in the equilibrium polymer and salt phases, which is confirmed by the identity of the electronic absorption spectra of the solutions. For example, the Co^2+^ ion in the initial solution of Co(NO_3_)_2_ and the equilibrium salt and polymer phases after extraction exists mainly in the form of an aqua complex. This is confirmed by the presence of an absorption band at 510 nm and a shoulder at 480 nm [32]. The absorption spectra of the initial solution of copper (II) nitrate and phases after extraction of Cu (II) are identical and have an absorption band at 800 nm, typical for [Cu(H_2_O)_6_]^2+^ [32]. The spectra of the initial solution of Ni(NO_3_)_2_ and the phases after extraction of Ni (II) are also identical and have absorption maxima at 393 and 721 nm and a shoulder at 657 nm related to the octahedral ion [Ni(H_2_O)_6_]^2+^. Thus, the distribution of metals in the studied extraction system is described by a simple physical distribution mechanism. The obtained dependence of the efficiency of metal extraction on the mixing time (Figure 1) is characteristic of the mechanism of physical distribution (Equation (1)) when the transition of the extracted component is quite fast.

(1) $${\left[Me{\left(H_{2}O\right)}_{x}\right]}_{\left(\mathrm{BP}\right)}^{n+}+kPEG_{\left(\mathrm{TP}\right)}↔{\left[Me{\left(H_{2}O\right)}_{x}{\left(PEG\right)}_{k}\right]}_{\left(\mathrm{TP}\right)}^{n+}\;$$

### 2.2. Extraction Isotherms of the Studied Metal Range in ATPS

**Figure 2:**
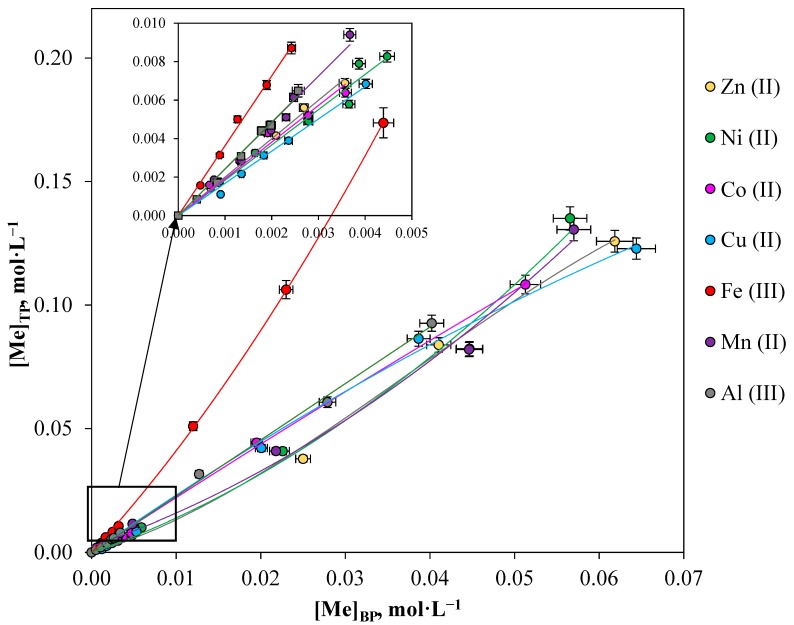
Metal extraction isotherms in the PEO 1500 (16.3 wt%)-NaNO_3_ (36.0 wt%)-H_2_O system: [Me]_in_ = 0–0.1 mol·L^−1^, temperature: 298.15 K, and phase ratio: 1:1.

The influence of the initial metal concentration was studied in the concentration range of between 0 and 0.1 mol·L^−1^. Figure 2 shows the isotherms of metal extraction in the PEO 1500 (16.3 wt%)-NaNO_3_ (36.0 wt%)-H_2_O system. The initial sections of the isotherms of metal extraction (insert in Figure 2) are of a straightforward nature. The straight-lined nature of these relationships proves that the metal distribution ratio is constant and does not depend on the initial metal concentration in the solution, which is important for simulating and implementing technogical processes. The angle of inclination of the extraction isotherm corresponds to the distribution ratio of the metal, its value, and the regression coefficient are presented in the Table 2.

As shown in Figure 2, metals were extracted at close distribution ratio values in a low concentration area. In the region of higher initial metal concentrations (more than 0.01 mol·L^−1^), the isotherms of metal extraction have an exponentially increasing character, which is characteristic of the mechanism of physical distribution.

### 2.3. Effect of Nitric Acid Concentration on the Metal Extraction in ATPS

**Figure 3:**
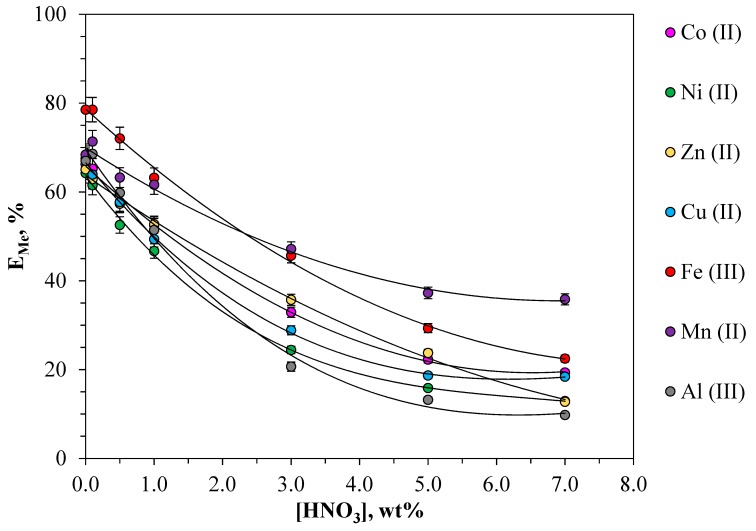
Effect of nitric acid on the metal extraction in the PEO 1500-NaNO_3_-H_2_O system: [Me]_in_ = 0.01 mol·L^−1^, temperature: 298.15 K.

Considering the fact that leaching solutions are mainly solutions with a high concentration of nitric acid (more than 1 mol·L^−1^), its concentration in the system is an important parameter which can influence both the metal ion form in the solution and the polymer phases degree of hydration. The influence of HNO_3_ acidity (in the range from 1 to 7 wt%) was studied for the first time on the extraction of a number of the metals studied in the PEO 1500-NaNO_3_-H_2_O system (Figure 3).

With an increase in the concentration of nitric acid from 1 to 7 wt%, a noticeable decrease in the degree of metal extraction in the studied extraction system is observed, for Mn(II) two times, for Fe(III), Cu(II), and Co(II) 3.5 times, for Ni(II) and Zn(II) five times, and for Al(III) almost seven times. The results showed that metals were better extracted in this system in the area of weakly acidic solutions. Introducing HNO_3_ into the system rendered it more difficult to transfer metal into the polymer phase due to the concurrent extraction of nitric acid. It should be noted that concentration of nitric acid affects phase separation and the ratio of phase volumes in the system. The more nitric acid there is in the system, the volume of the salt phase will increase. When the concentration of nitric acid is more than 7 wt%, the system becomes homogeneous.

### 2.4. Effect of the pH on Metal Extraction in ATPS

The increasing of salt stock solution acidity, in the pH domain of between zero and five, results in the decreasing of water content from PEO-rich phases. In this pH interval, none of phase-forming components (PEO, NO_3_^−^, Na^+^) are practically involved in secondary processes, and the increasing hydrophobicity of PEO-rich phase raise the number of extractants from extracted species [34,35].

**Figure 4:**
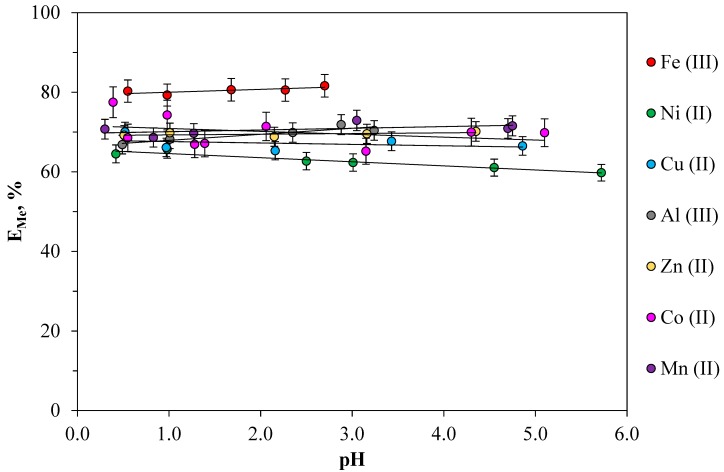
Effect of pH on the metal extraction in the PEO 1500 (16.3 wt%)-NaNO_3_ (36.0 wt%)-H_2_O system: [Me]_in_ = 0.01 mol·L^−1^, temperature: 298.15 K, and phase ratio: 1:1.

In the range of pH values from 0.5 to five, the effect of medium acidity on the extraction of a number of metals in the extraction system was studied. Figure 4 shows the relationships of the extraction of Fe(III), Co(II), Ni(II), Cu(II), Zn(II), Mn(II), and Al(III) in the PEO 1500 (16.3 wt%)-NaNO_3_ (36.0 wt%)-H_2_O system from the equilibrium pH values of the salt phase.

As can be seen from Figure 4, most metals (Co, Ni, Mn, Zn, Cu) are recovered in a wide range of pH values from 0.5 to five. In the studied pH ranges, the efficiency of metal extraction remains practically unchanged. This indicates that proton does not affect the extraction of metals. The studied pH range for each metal is determined by the pH value of the insoluble compounds formation in the heterogeneous system. The obtained dependences make it possible to determine the optimal pH value depending on a specific task. For example, in the case of complex extraction of all the studied metals, a pH range of 0.5 to two was chosen as the optimal pH value of the extraction system.

### 2.5. Effect of Temperature on Metal Extraction in ATPS

**Figure 5:**
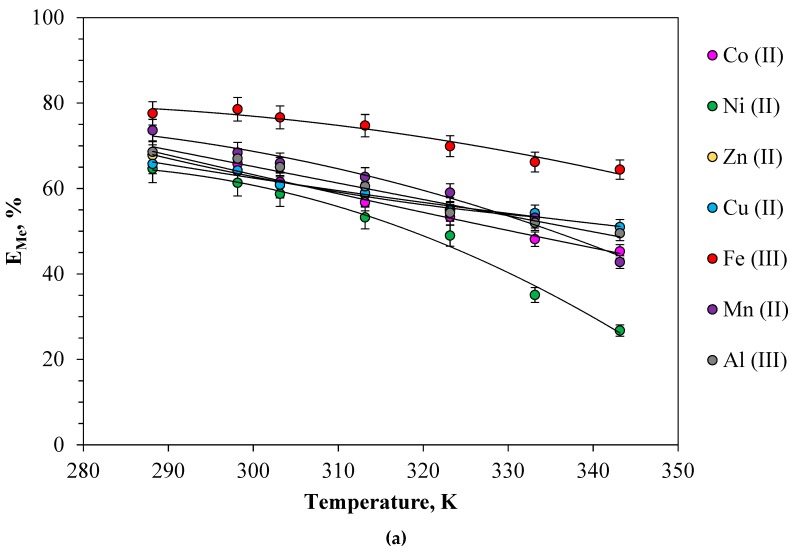

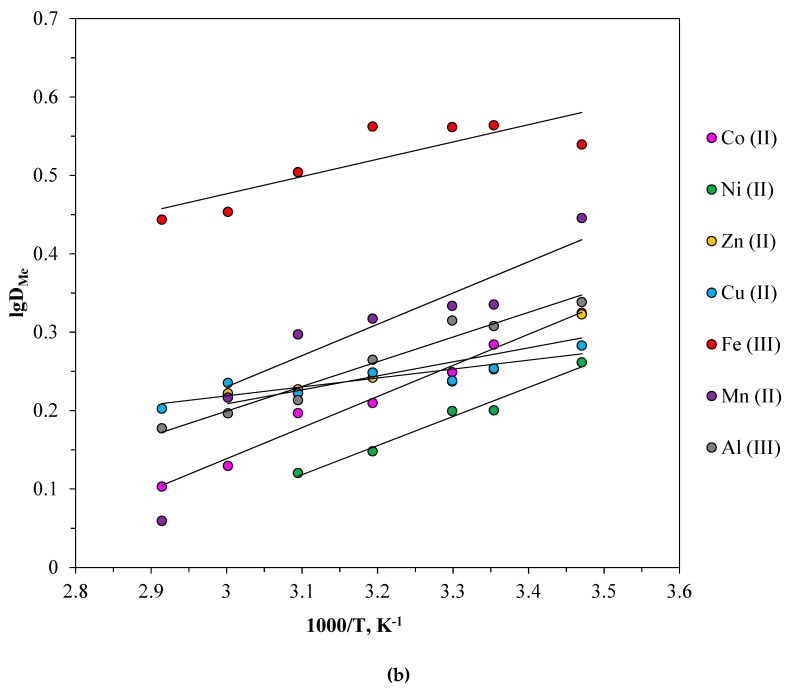
(**a**) Effect of temperature on the metal extraction; (**b**) plot of lgD_Me_ vs. 1000/T. Conditions: PEO 1500 (16.3 wt%)-NaNO_3_ (36.0 wt%)-H_2_O system, [Me]_in_ = 0.01 mol·L^−1^.

Temperature’s influence was studied on metal extraction in the 288.15–333.15 K range in the PEO 1500-NaNO_3_-H_2_O system. Figure 5a shows the effect of temperature on the extraction of metal salts from nitrate solutions. The results show that the temperature slightly affects the extraction efficiency in the range of 288.15–333.15 K, which is consistent with previously obtained data on the extraction of metal salts from chloride and sulfate solutions in systems with polyethylene glycol [36]. As can be seen in Figure 5a, the increase of liquid viscosity at low temperature might be not conducive to mass transfer. At an extraction temperature of 288.15 K, the maximum values of metal salts extraction efficiency were obtained, followed by slowly decrease with a further increase in the extraction temperature. This has to do with the fact that the process is exothermic and an increase in temperature does not favor extraction.

An increase in temperature leads to a change in the solubility of the components of the extraction system in each other, which is accompanied by a change in the ratio of phases and their composition. In the temperature range from 288.15 to 333.15 K, the solubility of PEO in water increases, which leads to the accumulation of PEO in the lower (enriched in sodium nitrates) phase. Therefore, this leads to the accumulation of metals, which also leads to a decline in the concentration of metals in the upper (rich in PEO) phase and, therefore, to a decrease in the rate of its extraction. In addition, an increase in temperature leads to energy consumption, which can increase production costs. Thus, low temperatures have a positive effect on metal extraction. We concluded that the optimal extraction temperature is 288.15–298.15 K.

In order to understand the extraction mechanism, it is helpful to obtain the thermodynamic parameters of the extraction because entropy and enthalpy changes could significantly affect the extent of the metal ion extraction equilibrium.

The enthalpy change of the extraction, ΔH, could be calculated from the slope of the lgD versus 1000/T by the Van’t Hoff Equation (2) [37,38]:

(2) $$lgD=-\frac{\Delta H}{2.303RT}+C\;$$

where *R* = 8.314 is the universal gas constant and *C* is a constant for the system.

The relationship between lgD and 1000/T in different solvents is shown in Figure 5b, and the calculated ΔH values are listed in Table 3. ΔH was similar to the bond energy of the hydrogen bond. The results show that ΔH < 0 in metals extraction, which indicated that the extraction process is exothermic. Therefore, the extraction experiment is suitable at room temperature.

The corresponding Gibbs free energy ΔG and entropy ΔS were calculated using Equations (3) and (4) [37], respectively, and their values were listed in Table 3.

(3) $$\Delta G=-RTlnK_{ex}\;$$

(4) $$\Delta S=\frac{\Delta H-\Delta G}{T}\;$$

As can be seen from Table 3, for all metals, ΔG are negative, therefore, the distribution ratio of metal ions is greater than unity. Moreover, the larger this value in absolute value, the more efficient the extraction, which is consistent with previously obtained data [38]. The variations in the changes of the thermodynamic properties of the extraction process may be due to the differences in the radii of the different ions.

**Figure 6:**
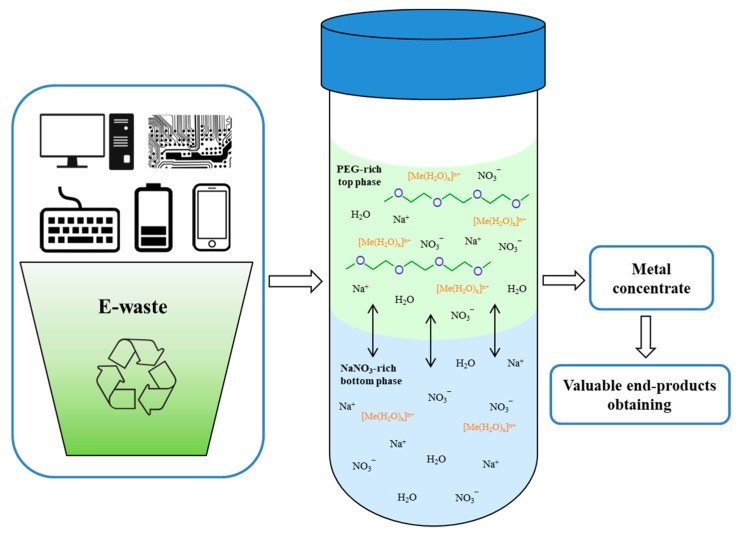
Scheme of the complex extraction of metal ions in the PEO 1500-NaNO_3_-H_2_O system and the possibility of its practical application.

Thus, this article proposes an extraction system based on polyethylene oxide 1500 without organic solvents and additional complexing agents for the complex extraction of Ni^2+^, Co^2+^, Fe^3+^, Mn^2+^, Zn^2+^, Cu^2+^, and Al^3+^ from nitrate solutions. Schematically, the proposed extraction mechanism и practical possibilities of these systems is shown in Figure 6. During the extraction process, metal aquacomlexes [Me(H_2_O)_x_]^n+^ connected with the polymer by hydrogen bonds are transferred into the polymer phase.

Electronic waste recycling will be a very important sector in the near future from economic and environmental perspectives. Recycling technology aims to take today’s waste and turn it into conflict-free, sustainable polymetallic secondary resources for tomorrow. Recycling technology must ensure that electronic waste is processed in an environmentally friendly manner, with high efficiency and lower costs. The proposed extraction systems can be promising in the development of hydrometallurgical processes for recycle of electronic waste.

## 3. Materials and Methods

### 3.1. Materials

The following chemicals were used in this study: Iron nitrate nonahydrate (Fe(NO_3_)_3_∙9H_2_O, ≥ 99.0%), manganous nitrate hexahydrate (Mn(NO_3_)_2_∙6H_2_O, ≥ 99.0%), copper nitrate 2.5-hydrate (Cu(NO_3_)_2_∙2.5H_2_O, ≥ 99.0%), cobalt nitrate hexahydrate (Co(NO_3_)_2_∙6H_2_O, ≥ 99.0%), nickel nitrate hexahydrate (Ni(NO_3_)_2_∙6H_2_O, ≥ 99.0%), zinc nitrate hexahydrate (Zn(NO_3_)_2_∙6H_2_O, ≥ 99.0%), aluminum nitrate nonahydrate (Al(NO_3_)_3_∙9H_2_O, ≥ 99.0%), sodium nitrate produced by Sigma-Aldrich (ACS chemical, ≥ 99.0%, St. Louis, MO, USA) (CAS no. 7631-99-4), 4-(2-pyridylazo)resorcinol (PAR) (CAS no. 1141-59-9), xylenol orange (CAS no. 3618-43-7), nitric acid (chemically pure), and poly(ethylene oxide) with a molecular weight of 1500 produced by Sigma-Aldrich (CAS no. 25322-68-3).

Double distilled water was used to prepare the solutions. All chemicals were used as received, without additional purification. The initial solutions of extracted metals nitrates were obtained by dissolving precisely weighed quantities in double distilled water slightly acidized with nitric acid (pH = 2.5) in order to prevent the generation of hydroxide metal formations.

### 3.2. Extraction Studies

All extraction experiments were carried out at a temperature of 298.15 K (excluding temperature relationships) and an atmospheric pressure of ~100 kPa in a temperature-controlled shaker (the temperature range was 277.15–348.15 K and the accuracy was ± 0.2 K) (Enviro-Genie SI-1202, Scientific Industries, Inc., Bohemia, NY, USA).

To create the extraction system (poly(ethylene oxide) 1500 (16.3 wt%)-sodium nitrate (36.0 wt%)-water), polymer, salt, and aliquot of extracted metal were dissolved in double distilled water and mixed until two-phase equilibrium was generated. All the experiments were conducted in graduated centrifugal test tubes (15 mL). The chemicals were weighed on an analytical balance with an accuracy of ±0.0001 g (HR-100AZ, AND Company, Seoul, Korea). Then, an aliquot of extracted metal salt acidified with nitric acid and a 0.1 mol·L^−1^ (pH = 2.5) concentrated were added to the created aqueous two-phase system.

The test tubes were placed into the temperature-controlled shaker and intensely mixed at constant temperature over the course at a rotation speed of 30 rpm to reach the thermodynamic equilibrium (see. Figure 1). To determine the time to establish thermodynamic equilibrium, the phase contact time was varied from 5 to 60 min. Then, the samples were centrifuged at 2500 rpm for 10 min to achieve complete separation in the centrifuge (CM-6MT, SIA ELMI, Riga, Latvia). The volumes of the top and bottom phases were measured as well. Then, the phases were separated and the pH equilibrium values and concentration of metal ions were determined in both phases.

The concentration of metals in the initial solution in the equilibrium polymer and salt phases after the extraction was performed using the spectrophotometric method based on obtaining complexes of metals with 4-(2-pyridylazo)resorcinol absorbing in the visible spectrum had the following wavelengths, nm: 492 (Ni), 508 (Co), 420 (Fe), 496 (Mn), 490 (Zn), and 494 (Cu) [39] in the Cary-60 spectrophotometer (Agilent Tech., Santa Clara, CA, USA) (wavelength accuracy ± 0.06 nm). The concentration of aluminum was also determined spectrophotometrically using xylenol orange at pH = 3.5 and a wavelength of 550 nm [40]. The concentration determination error was < 5%.

Studying the influence of the concentration of nitric acid on the distribution of metal salts, the contents of nitric acid were varied from 1 to 7.0 wt% and sodium nitrate to the system so that the total concentration of nitrate ions in the system remained constant at 36.0 wt%.

The isotherms of extraction of metal salts were obtained by varying the concentration of metal salts in the system from 0 to 0.1 mol·L^−1^.

When building relationships between metal ion extraction and acidity, the required pH-value was reached by adding nitric acid which was controlled with an accuracy of ± 0.001 by a pH-meter (Starter 5000, OHAUS, Parsipanny, NJ, USA) with a combined STMICRO5 RU glass electrode calibrated against buffers with the pH-values 1.68, 4.01, 7.00, and 10.01 (at 298.15 K).

The temperature levels for metal ion extraction were taken in the 288.15–333.15 K temperature range.

For the purpose of the quantitative description and efficiency estimation of metal ions’ extraction recovery, the distribution ratio (D_Me_) and extraction efficiency (E_Me_, %) were used, which were determined by Equations (5) and (6):

(5) $$D_{\mathrm{Me}}=\frac{{\left[\mathrm{Me}\right]}_{\mathrm{TP}}}{{\left[\mathrm{Me}\right]}_{\mathrm{BP}}}\;$$

(6) $$E_{\mathrm{Me}}=\frac{{\left[\mathrm{Me}\right]}_{\mathrm{TP}}\cdot V_{\mathrm{TP}}}{{\left[\mathrm{Me}\right]}_{\mathrm{BP}}\cdot V_{\mathrm{BP}}+{\left[\mathrm{Me}\right]}_{\mathrm{TP}}\cdot V_{\mathrm{TP}}}\times 100\;\%$$

where ${\left[\mathrm{Me}\right]}_{\mathrm{TP}}$ is the metal concentration in the top phase, mol·L^−1^, ${\left[\mathrm{Me}\right]}_{\mathrm{BP}}$ is the metal concentration in the bottom phase, mol·L^−1^, and $V_{\mathrm{TP}}$ and $V_{\mathrm{BP}}$ are the volumes of the top and bottom phases, respectively.

All experiments were repeated three times and the measurement error was < 5%.

## 4. Conclusions

The novel eco-friendly extraction PEO 1500 (16.3 wt%)-NaNO_3_ (36.0 wt%)-H_2_O system for complex recovery of Ni^2+^, Co^2+^, Fe^3+^, Mn^2+^, Zn^2+^, Cu^2+^, and Al^3+^ ions from nitrate solutions was suggested. The influence of various system parameters, such as medium acidity, temperature, metal initial concentration, contact time of phases on metal extraction behavior was studied. The advantage of the proposed system in comparison to other systems based on polymers is the possibility of extraction without additional complexing agents. It was established that in the studied system all metals are extracted, with more than 60% in one extraction step. The metal ions extraction mechanism was proposed. It was established that metals aquacomlexes, which are connected with the polymer by hydrogen bonds are transferred into the polymer phase. The linear nature of the extraction isotherms indicates the similarity of the metal ions distribution coefficients, which is important for subsequent technological processes development. It was shown that adding HNO_3_ into the system negatively influence the recovery, due to competing acid extraction into the polymer phase. The thermodynamic parameters of metal ions extraction were calculated. It was established that the studied process is exothermic, due to which the higher metal ions extraction achieved at room temperatures. The possibility of using of aqueous two-phase systems based on poly(ethylene oxide) 1500 and sodium nitrate for the effective extraction of a number of metals from nitric solutions has been shown.

Thus, an alternative technique that can replace traditional liquid–liquid extraction operations has been proposed in this work for the extraction Ni^2+^, Co^2+^, Fe^3+^, Mn^2+^, Zn^2+^, Cu^2+^, and Al^3+^ ions. The technique employs organic solvent free systems that are based on the principles of green chemistry, with good efficiency and economic viability. The proposed extraction systems can be promising in the development of chemical-technological processes for recycle of electronic waste to achieve the objectives of “a minimization of waste” and “the stable recovery of resources”. However, further work is necessary to improve this technique for industrial applications.

## Figures and Tables

**Table 1:** The initial composition, the composition of the top and bottom phases for the system based on PEO 1500 and NaNO_3_ and Na_2_SO_4_ at 298.15 K (compositions in wt%) and ~100 kPa.

System	Overall			Top Phase			Bottom Phase		
	PEO	Salt	Water	PEO	Salt	Water	PEO	Salt	Water
PEO 1500-Na_2_SO_4_-H_2_O [33]	15.0	9.0	76.0	26.7	4.1	69.2	0.6	16.8	82.6
PEO 1500-NaNO_3_-H_2_O [28]	16.3	36.0	47.7	31.6	27.0	41.4	1.1	45.6	53.3

**Table 2:** Slope and the values of the regression coefficient for the initial linear section ([Me]_in_ = 0–0.01 mol·L^−1^) of metal extraction isotherms in the PEO 1500 (16.3 wt%)-NaNO_3_ (36.0 wt%)-H_2_O system.

Metals	Slope	R^2^
Fe(III)	3.6275	0.9962
Co(II)	1.9072	0.9694
Ni(II)	1.8347	0.9720
Cu(II)	1.6732	0.9930
Zn(II)	1.9961	0.9955
Mn(II)	2.4156	0.9851
Al(III)	2.4284	0.9931

**Table 3:** Extraction of metals thermodynamic function values.

Metal	Slope	∆H (kJ/mol)	∆G (kJ/mol)	∆S (J/mol·K)
Co(II)	0.3969	−7.55	−1.35	−20.15
Ni(II)	0.3713	−6.81	−0.77	−19.77
Zn(II)	0.1787	−3.37	−1.46	−6.27
Cu(II)	0.1139	−2.16	−1.44	−2.35
Mn(II)	0.3989	−7.52	−1.88	−18.50
Fe(III)	0.2203	−4.17	−3.09	−3.53
Al(III)	0.3150	−5.94	−1.58	−14.28
